# Irritability Moderates the Association between Cognitive Flexibility Task Performance and Related Prefrontal Cortex Activation in Young Children

**DOI:** 10.3390/brainsci13060882

**Published:** 2023-05-31

**Authors:** Yanwei Li, Adam S. Grabell, Susan B. Perlman

**Affiliations:** 1 College of Early Childhood Education, Nanjing Xiaozhuang University, Nanjing 210017, China; 2 Department of Psychological and Brain Sciences, University of Massachusetts, Amherst, MA 01003, USA; 3 Department of Psychiatry, Washington University in St. Louis, St. Louis, MO 63130, USA

**Keywords:** cognitive flexibility, prefrontal cortex, irritability, moderation, early childhood

## Abstract

The association between cognitive flexibility and related neural functioning has been inconsistent. This is particularly true in young children, where previous studies have found heterogenous results linking behavior and neural function, raising the possibility of unexplored moderators. The current study explored the moderating role of dimensional irritability in the association between cognitive flexibility task performance and prefrontal activation in young children. A total of 106 3- to 7-year-old children were recruited to complete a custom-designed, child-adapted, cognitive flexibility task, and 98 of them were included in the data analysis. The children’s dorsolateral prefrontal cortex activation was monitored using functional near-infrared spectroscopy, and their levels of irritability were reported by parents using the MAP-DB Temper Loss subscale. Results indicated that the mean reaction time of the cognitive flexibility task was negatively correlated with concurrent prefrontal activation. No evidence was found for the association between task accuracy and prefrontal activation. Moreover, irritability moderated the association between the mean reaction time and prefrontal activation. Children high in irritability exhibited a stronger negative association between the mean reaction time and related prefrontal activation than children low in irritability. The moderating model suggested a novel affective–cognitive interaction to investigate the associations between cognitive task performance and their neural underpinnings.

## 1. Introduction

Cognitive flexibility, defined as the ability to simultaneously process multiple demands in order to switch between them and respond appropriately [1], has been demonstrated to be critical for an individual’s lifelong development [2]. It is a significant predictor for the development of higher-order cognition, such as academic achievement [3,4], creativity [5], decision-making [6], and problem-solving [7]. Cognitive flexibility has also been demonstrated to enhance children’s social [8] and emotional competence [9], with optimal outcomes in adulthood, such as higher life satisfaction [10] and higher resilience to negative life experiences [11]. Deficits in cognitive flexibility are common symptoms of neurodevelopmental disorders such as attention deficit hyperactivity disorder and autism spectrum disorder as well as mood and anxiety disorders [2]. Emerging evidence from neuroimaging studies has highlighted the role of the prefrontal cortex in cognitive flexibility, and research has begun to explore how individual factors, such as personality traits, may influence the neural correlates of cognitive flexibility.

Given its critical role in human development, recent studies have investigated the neural underpinnings of cognitive flexibility in adults and children. The prefrontal cortex (PFC) is generally considered a hub for cognitive flexibility and related behaviors with activation extend to the ventral and lateral PFC [12]. For example, using a classic color-word Stroop task, one functional magnetic resonance imaging (fMRI) study in adults found that bilateral prefrontal cortex regions were activated for the contrast between incongruent and neutral conditions [13]. Similarly, 8- to 12-year-old children showed bilateral prefrontal activation during a numerical Stroop task when faced with the inconsistency between the font size of a number and its numerical value [14]. The developing prefrontal cortex was also found to be related to persistent errors during the Dimensional Change Card Sorting Task used to evaluate cognitive flexibility in preschool-age children [15,16]. Using functional near-infrared spectroscopy (fNIRS), our previous studies found activation in the lateral PFC regions when comparing the concurrent and non-concurrent conditions during a child-friendly Stroop task in children aged 3–5 [17,18]. Taken together, these studies and other studies in the field highlight the importance of the PFC in the development of cognitive flexibility using multiple imaging modalities.

One question that remains in current cognitive flexibility research is the association between PFC activation and task performance while the brain is engaged in executive function tasks. Some researchers have reported a positive correlation between PFC activation and cognitive flexibility task performance. For example, previous studies found that the right lateral and posterior orbital gyri were activated while adults shifted their response according to feedback during a reversal learning task [19]. Furthermore, the activation in the right inferior frontal gyrus of the PFC was positively related to a change in post-reversal accuracy. Surprisingly, though, many studies fail to find the expected association between brain activation and behavior. For example, using fNIRS to explore the role of the prefrontal lobe during a switch task, some researchers found that the frontopolar area and left frontal eye fields were significantly activated while healthy adults flexibly changed their judging rules to identify the odd/even or low/high property of numbers from 0 to 9 [20]. However, the oxy-hemoglobin concentration was not related to the switch task performance. Additionally, using fNIRS to investigate the neural underpinning of the cognitive set-shifting task, researchers found the frontal area was significantly activated while healthy adults drew a line alternately and flexibly shifted between consecutive numbers 1 and 12 and letters A and L [21]. However, there was no significant association between PFC activation and task performance. These studies were conducted only in adults, and the contradictory findings might be caused by different experimental task designs or variability in specific regions of interest in the PFC. However, individual differences in person-specific features, such as personality traits, might be another explanation for conflicting findings between neural activation and cognitive flexibility task performance, which has been much less explored, especially in young children, in the current literature.

Numerous reviews have demonstrated that personality or temperament characteristics are associated with PFC activation critical for cognitive functions [22,23,24]. One such domain, irritability, defined as a prolonged mood state characterized by negative responses (i.e., anger, tantrums, or frustration) to blocked goals [25], has more recently been tied to variability in cognitive functioning [26,27,28]. Irritability, which varies in terms of context, quality, and intensity, falls along the normal to clinically impaired spectrum [29]. Increased irritability has been linked to poor cognitive flexibility task performance [30] and incapacity to adjust to altering environmental demands [31]. Along with behavior, the relationship between irritability and cognitive flexibility has been identified at the neural level [32,33,34,35]. For example, using episodic irritability-eliciting tasks (i.e., tasks that induce frustration), increased prefrontal activation has been found in adults [36] and children [34,37,38], which has been related to elevated irritability [34,39,40]. Lesion studies provide additional evidence that prefrontal regions are critical for managing irritability. Subjects with prefrontal brain trauma have been noted to have poor irritability regulation, described as exhibiting short temper and anger [41,42].

Multiple studies have delineated the affective–cognitive interaction, specifically that of cognitive flexibility and irritability, at the neural level. For example, our previous study found that increased cognitive flexibility-related prefrontal activation was related to elevated levels of irritability in preschool children, suggesting a positive association between irritability and cognitive tasks [18]. This study was conducted in a typically developing sample; however, other studies investigating clinically impaired individuals with severe irritability have found less prefrontal engagement (i.e., reduced prefrontal activation) during cognitive tasks. For instance, using a reversal learning task to measure cognitive flexibility, a prior study found that clinically impaired adolescents with severe chronic or episodic irritability exhibited less cognitive flexibility-related prefrontal activation than typically developing controls [43]. These findings indicate that the association between cognitive flexibility-related brain activation and the full spectrum of normal to abnormal irritability might vary along the normative to clinical irritability trajectory. For the normative range of irritable temperament, PFC activation and irritability may correlate positively during cognitive flexibility tasks; however, for children with clinically salient irritability, cognitive flexibility-related activation might be lower than that of the typically developing but temperamentally irritable subjects. Collectively, the abovementioned findings suggest that PFC is a hub of cognitive flexibility. These findings indicate that both cognitive flexibility and irritability may share common neural substrates and mutually affect individuals’ developmental processes. In line with previous findings indicating that executive function training may be an effective intention to ameliorate psychological adjustment [44,45], we aimed to investigate whether cognitive flexibility would be a potential target to reduce irritability. If so, how and for whom would the intervention be effective?

Extant literature suggests an interaction between cognitive flexibility and irritability at the behavioral level. For example, a longitudinal, population-based study found that the associations between childhood-onset irritability and delinquency (a latent variable including psychopathology, self-report of delinquent behaviors, and criminal records) in young adulthood varied based on the development of cognitive flexibility [46]. Compared to peers with high childhood-onset irritability but lower cognitive flexibility during adolescence, boys with higher adolescent cognitive flexibility exhibited less severe delinquency rates. Few studies, however, have investigated the moderating role of irritability in direct relation to neural underpinnings of cognitive flexibility, especially for young children. Thus, we aimed to examine the potential moderating role of irritability on the association between cognitive flexibility task performance and PFC activation during early childhood.

However, the lack of consistent findings in the literature and the potential influence of individual factors on this relationship pose significant challenges to understanding the neural mechanisms underlying cognitive flexibility, limiting the generalizability of previous findings and the development of effective interventions for individuals with cognitive impairments. Therefore, the present study aims to investigate the relationship between prefrontal activation and cognitive flexibility task performance, taking into account the potential moderating effect of irritability, a trait associated with both cognitive flexibility and PFC activation, in a normative sample of children. We used functional near-infrared spectroscopy (fNIRS) to examine the association between performance on a child-friendly cognitive flexibility task and concurrent PFC activation in a sample that included children recruited from the community and local psychiatric clinics. Consistent with previous research [20], we hypothesized that children who performed better during the task would recruit more neural support from the PFC. Thus, we expected that higher levels of PFC activation would be associated with high accuracy or low mean reaction time in the cognitive flexibility task. Next, we tested the moderating role of irritability in this association. We hypothesized that irritability would have a moderating effect on the association between cognitive flexibility task performance and PFC activation. Based on our previous finding that higher levels of irritability were associated with increased PFC activation [18], we postulated that children high in irritability would engage more PFC activation when compared with children low in irritability during the cognitive flexibility task. Thus, the association between cognitive flexibility task performance and PFC activation would be stronger for children with higher levels of irritability.

## 2. Materials and Methods

This study was approved by the Institutional Review Board (IRB) of the University of Pittsburgh.

### 2.1. Participants

In order to increase the number of high irritability children in our sample, both community and clinical children were recruited to participate. The community-recruited children were recruited via flyers and local advertisements. They were reported by their parents to have no history of psychiatric diagnoses during their lifetime nor to have any psychiatric disorders associated with their first-degree relatives and no history of head trauma with loss of consciousness. The clinic-recruited children were recruited via flyers, through an online registry, and by clinic staff if they were seeking assessment or intervention services at an outpatient clinic. Exclusion criteria in clinically-recruited children included developmental disorders, psychotic disorders, neurological disorders, and a history of head trauma with loss of consciousness.

One hundred and six (58 boys) 3- to 7-year-old children (mean = 71.25 months, SD = 15.99, range = 38–96.00 months) were recruited to participate in the present study, with 78 community-recruited and 28 clinic-recruited participants. Of these children, 68 were Caucasian, 36 were African–American, and 2 were Asian. The median family income was $55,000 (range: $6000–$1,200,000).

### 2.2. Cognitive Flexibility Task

**Figure 1:**
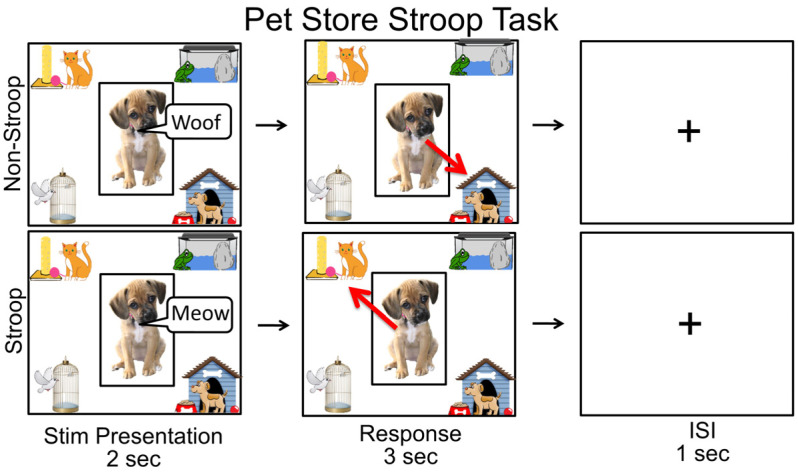
Pet Store Stroop Task. In the non-Stroop condition, children were instructed to put the animal (i.e., dog) in the correct cage (i.e., the dog’s cage) according to the animal’s appearance; in the Stroop condition, children were instructed to put the animal (i.e., cat) in the correct cage (i.e., the cat’s cage) according to the sound it made.

A custom-designed, child-friendly Pet Store Stroop Task (see Figure 1) [18], modified based on the classic Stroop paradigm [47], was used to evaluate the children’s cognitive flexibility. Children were told the story of a pet shop in which all the animals escaped from their cages. They were asked to put each escaped animal back into the correct cages by touching the targeted cages. The animals would appear in the center of the screen and make a sound (2 s). Children were asked to touch the correct cage, which appeared in the corners of the screen (i.e., dog into the doghouse) in the following 3 s. In the non-Stroop, control condition, the sound that the animal made matched its appearance (i.e., the dog says “woof”). Whereas, in the Stroop condition, the child was told that the animals sometimes disguised themselves as other animals, so they must listen carefully for the sound that the animal makes to tell them what the real animal was (i.e., the dog says “meow”, so it is really a cat). Therefore, children had to inhibit their predominant response to sort out the animals according to their appearance, but instead sort the animals based on their incongruent sounds. The task contained three Stroop and three non-Stroop blocks in interchanging order, with 6 trials in each block and a total of 18 trials for each Stroop/non-Stroop condition. There was a fixation cross for one second between each trial and for fifteen seconds between each block. The entire task lasted about five minutes. Children were seated at a child-sized desk in front of a touch screen computer and completed the task. One experimenter sat with the child and explained the task while another experimenter monitored the fNIRS data acquisition. The whole session would take about 10 min. Accuracy (ACC) and mean reaction time (RT) extracted from the corrected trials were utilized to represent the task performance.

### 2.3. fNIRS Data Acquisition and Preprocessing

A CW6 real-time fNIRS system was used for imaging data acquisition (Techen, Inc., Milford, MA, USA). A child-friendly elastic cap was designed to measure the prefrontal hemoglobin activation by using a customized fNIRS cap. The fNIRS probe, consistent with our previous study [18], comprised 4 light-source positions, each containing 690 nm (12 mW) and 830 nm (8 mW) laser lights, and 8 detectors covering the dorsolateral prefrontal cortex, specifically Brodmann areas 10 and 46 on each hemisphere. An LPT port was used to synchronize the stimulus timing from E-Prime (Psychology Software Tools, Sharpsburg, PA) with prefrontal hemoglobin changes.

fNIRS raw signals were recorded as dynamic light changes from a source position incident on a detector position and were collected at 20 Hz by using a custom-built Matlab-based (Mathworks, Natick, MA, USA) acquisition software program and the NIRS Toolbox (http://huppertlab.net/nirs-toolbox-2/, accessed on 18 October 2015). Real-time visualization for the fNIRS signals (oxy- and deoxy-hemoglobin) during collection was checked by the operator. Meanwhile, events such as motion or distractions were added to the fNIRS data in real-time to indicate if the subject focused on the task. The duration of acquisition is about 5 min. Data was then Nyquist filtered and down-sampled to 4 Hz (Time intervals = 250 milliseconds) before analysis [48]. Consistent with previous fNIRS research [49], the light changes were transformed to optical density and hemoglobin concentration changes through the modified Beer–Lambert law [50], with a differential path length term of six for both wavelengths [51]. The dynamic hemoglobin changes were then corrected for motion-related artifacts via an autoregressively whitened robust regression model [48], which has good control of the type I error (false-discovery rate) [52]. In brief, a general linear model (GLM, e.g., *y* = *Xβ* + *ε*) was constructed, where *X* was defined as the design matrix and *β* was defined as the magnitude of brain activation in the evoked hemodynamic response. An iterative auto-regressive (AR) filtering was performed to remove the effect of serial correlations in the residual of the GLM model via applying a linear whitening filter (*S*^−1^) on both sides of the equation (e.g., *S*^−1^·*y* = *S*^−1^·*Xβ* + *S*^−1^). The estimated regression coefficients (*β*) for each condition and each channel at the subject level were used to conduct statistical tests at the group level. The cognitive flexibility task-related brain activation was characterized as the relative changes of brain activation between Stroop and non-Stroop blocks (*β* = *β*_Stroop_ − *β*_Non-Stroop_). Oxy-hemoglobin changes were reported to have a higher signal-to-noise ratio [53] and a stronger correlation with the BOLD signal [54] than the deoxy-hemoglobin changes. Thus, in the current study, we used the oxy-hemoglobin results to estimate the associations with cognitive flexibility task performance and irritability.

### 2.4. Parent-Rated Irritability

The children’s irritability was reported by their parents via the Temper Loss subscale of the Multidimensional Assessment Profile for Disruptive Behavior (MAP-DB) [55]. This subscale was developed to discriminate the full dimension of normative to clinically salient irritability, including irritability behaviors such as tantrums and irritable mood [56,57]. It comprised 22 items (e.g., “Yell angrily at someone”, “Have a hot or explosive temper”) rated on a 6-point Likert scale (0 = “Never”, 5 = “Many times each day”). The reliability of the subscale was excellent (α = 0.96).

### 2.5. Analysis Strategy

A priori power analyses conducted in G*Power indicated that a total sample size of N = 89 was needed to detect a medium effect size of 0.15 [58]. Taking into consideration that some of the children might not understand our task or exhibit poor-quality fNIRS data, a total of 106 children were recruited to participate in the current study. We excluded eight participants from the dataset due to low accuracy or outliers in PFC activation. Six children were excluded because their accuracy was below the rate of success from guessing, which was 25% in the current study, in either Stroop or non-Stroop conditions. Two children were excluded because their mean PFC activation values exceeded the range of ± 3SD from the mean activation of all participants. Thus, ninety-eight participants (*Mean* age = 71.45 months, *SD* = 15.95, *Range* = 38–96.00 months) were included in the data analysis. Of these children, 52 were boys and 46 were girls; 71 were community-recruited and 27 were clinic-recruited. Since the current study was designed to test a dimensional irritability sample, all children were included in the data analysis as a whole sample.

Data analyses were performed via the SPSS-21 software (IBM), and the moderating effect of irritability was probed by the PROCESS-3.4 macro (Model 1) [59]. First, a paired-sample *t*-test was conducted to test the behavioral performance difference between Stroop and non-Stroop conditions. Second, a bivariate correlation was performed to test the associations between task performance and PFC activation at the channel level. As we have performed in previous studies [60,61], our strategy was to combine significant channels into a region of interest. We then used the mean activation of this ROI to investigate PFC activation during the task. Third, using Model 1 of PROCESS 3.4 macro, a multiple regression analysis was conducted, with performance as the predictor, PFC activation as the dependent variable, and irritability as the moderator. Regression coefficients for the effects of the predictor, moderator, and the interaction term predictor × moderator were calculated.

## 3. Results

### 3.1. Cognitive Flexibility Task Performance

PAIRED-sample *t*-tests were conducted to test whether accuracy and mean reaction time differed between the Stroop and non-Stroop conditions. As expected, children were less accurate (Stroop: *Mean* = 0.75, *SD* = 0.19, non-Stroop: *Mean* = 0.79, *SD* = 0.18; *t* (97) = −3.60, *p* < 0.001) and slower (Stroop: *Mean* = 2.79 s, *SD* = 0.49, non-Stroop: *Mean* = 2.56 s, *SD* = 0.47; *t* (97) = 6.74, *p* < 0.001) in the Stroop condition than in the non-Stroop condition, suggesting that the participants, in general, performed better in the non-Stroop condition than in Stroop condition.

### 3.2. Association between the Cognitive Flexibility Task Performance and the PFC Activation

**Figure 2:**
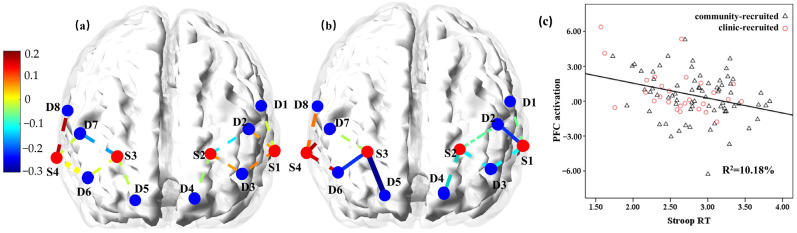
Associations between Stroop task performance and PFC activations. (**a**) Presents the channel space map showing the association between the Stroop accuracy and PFC activations; (**b**) presents the channel space map showing the association between Stroop mean reaction time and PFC activations. Solid lines denote significant correlations, whereas dotted lines denote non-significant correlations. (**c**) is the scatter plot of the relationship between Stroop mean reaction time and PFC activations. Black triangles denote community-recruited participants, and red circles denote clinic-recruited participants.

A bilateral correlation analysis was conducted to test the association between task performance and PFC activation. Stroop accuracy was unrelated to the PFC activation in all twelve channels (*p* values > 0.05; see Figure 2a). However, as shown in Figure 2b, the Stroop mean reaction time (Stroop RT) was significantly negatively related to the activations in channels (source-detector) S1-D2, S3-D5, and S3-D6 (*r* values < −0.22, *p* < 0.05). Thus, these channels were combined into regions of interest, and their mean activation was used to represent PFC activation during tasks. The PFC activation was related to Stroop RT with *r* = −0.32 and *p* = 0.001. A scatter plot of the relationship between Stroop RT and the mean PFC activation during the cognitive flexibility task is shown in Figure 2c.

### 3.3. The Moderating Role of Irritability in the Association between the Cognitive Flexibility Task Performance and PFC Activation

Parents reported a range of irritability scores (*Mean* = 23.60, *SD* = 19.95, *Range* 0–107; maximum possible score = 110). Based on irritability scores higher than 35.7, corresponding to the 90th percentile of the community sample (Wakschlag, unpublished data), 80.6% of the current sample was in the normative range, and 19.4% was in the clinically significant range. Moreover, the distribution of the irritability scores in the clinic-recruited children (*Mean* = 31.80, *SD* = 19.12, *Range* 0–107) overlapped with that in the community-recruited children (*Mean* = 20.48, *SD* = 19.50, *Range* 1–70), comprising a wide range of low to high irritability scores. An independent *t*-test revealed that clinic-recruited children exhibited significantly higher irritability scores than community-recruited children (*t*(96) = 2.58, *p* = 0.01).

The bilateral correlation analysis revealed that the irritability scores were not related to Stroop RT or Stroop ACC (*p* values > 0.05).

Next, to test whether irritability moderates the association between task performance and PFC activation, a moderation model was conducted via PROCESS-3.4 macro for SPSS (Model 1) with all variables standardized. Since Stroop accuracy was not associated with the PFC activation, Stroop RT was utilized in the model as the predictor. The interaction term (Stroop RT × irritability) was not significant (*p* > 0.05) when using the ROI activation as the dependent variable. Instead, based on previous research [17], the most significantly correlated channel (S3-D5), which was still significant after multiple comparison corrections (false discovery rate corrected, *r* = −0.30, *p _corrected_* = 0.034), was used to represent the PFC activation. We did not utilize age and gender as covariates because they were unrelated to PFC activation (*p* values > 0.05). The moderation model was significant at *F* (3, 94) = 6.58 (*p* < 0.001). Stroop RT, irritability, and Stroop RT × irritability explained 17.37% of the variance in the PFC activation. Additionally, Stroop RT negatively predicted (*B* = −0.35, *p* < 0.001) and irritability positively predicted (*B* = 0.28, *p* < 0.01) PFC activation. The addition of the two-way interaction Stroop RT × irritability negatively predicted the PFC activation (*B* = −0.22, *p* = 0.038), explaining 3.91% of the variance in the PFC activation (see Table 1). The significant interaction term indicated that different levels of irritability had different implications for associations between Stroop RT and PFC activation.

**Figure 3:**
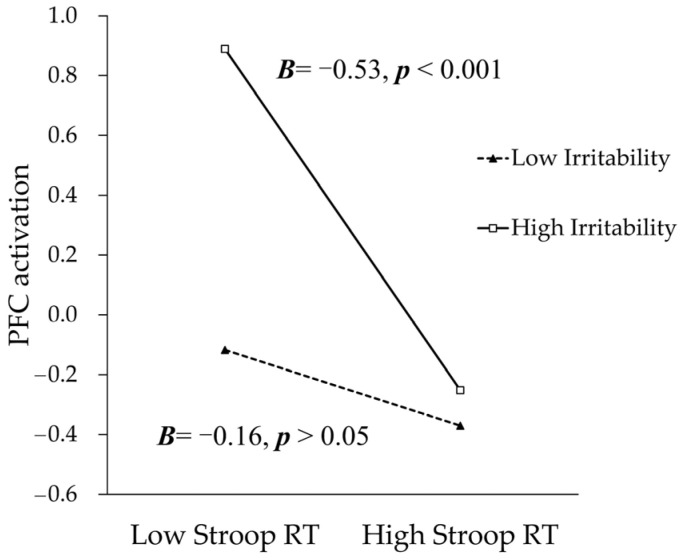
The interaction effects between Stroop RT and child irritability in predicting PFC activations. Low irritability represents 1 SD below the mean, and high irritability represents 1 SD above the mean. Low Stroop RT represents 1 SD below the mean, and high Stroop RT represents 1 SD above the mean. Solid lines denote significant simple slopes, whereas dotted lines denote non-significant simple slopes.

As shown in Figure 3, the association between Stroop RT and the PFC activation was stronger for children with higher levels of irritability. For children with low levels of irritability (−1 SD below the mean), the Stroop RT was not significantly linked with PFC activation (*Simple slope* = −0.16, *t*(94) = −1.29, *p* > 0.05). For children with high irritability (+1 SD above the mean), the Stroop RT was significantly and negatively associated with PFC activation (*Simple slope* = −0.53, *t*(94) = −3.99, *p* < 0.001), suggesting that highly irritable children utilize more PFC activation to respond faster during the cognitive flexibility task.

**Figure 4:**
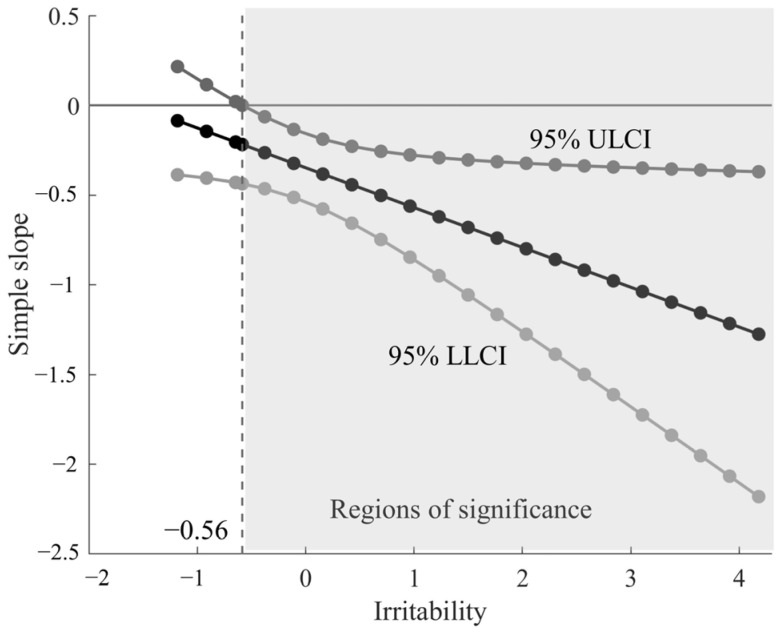
Johnson–Neyman plot of the conditional effect of Stroop mean reaction time on PFC activations across irritability. Note: 95% ULCI represents the upper level of 95% confidence interval, and 95% LLCI represents the lower level of 95% confidence interval. Regions of significance are marked in light gray.

The Johnson–Neyman plot (Figure 4) presents a simple slope for the moderator from non-significant to significant. It shows that the effect of Stroop RT on the PFC activation was completely significant for children’s irritability z-scores above −0.56. The regions of significance analysis showed that the effect of Stroop RT on the PFC activations was enhanced with increasing irritability, indicating an irritability-related negativity effect in the relationship between Stroop mean reaction time and PFC activation.

## 4. Discussion

In the current study, we found a negative association between the mean reaction time of the cognitive flexibility task and the related PFC activation, which was moderated by early childhood irritability. The results indicated that children who responded faster during the cognitive flexibility task recruited more PFC activation and that such association was stronger for children with higher levels of irritability. The current study enriches our understanding of how irritability and cognitive flexibility may be intertwined at the neural level. It is among the first to demonstrate that individual differences in personality or temperament moderate the association between cognitive flexibility task performance and its neural underpinnings. The moderation model provides evidence that heterogenous results on the associations between behavioral task performance and co-occurring neural functioning could be influenced by the personality traits of the participants. This finding contributes to our understanding of how the cognitive–affective processing system develops at the neural level during early childhood.

Inconsistent with previous studies [20,21,62,63], our findings found that increased PFC activation was associated with decreased mean reaction time on the cognitive flexibility task. This suggests that increased PFC activation is positively correlated with better behavioral performance. Thus, our results fall in line with findings that better task performance is associated with increased activation in brain regions responsible for information processing [19,64]. Our finding that PFC activation was associated with the cognitive flexibility task adds to the growing body of research on the role of the PFC as a “hub” for young children’s cognitive flexibility development.

Additionally, we employed a moderation model and found that early childhood irritability moderated the association between cognitive flexibility task performance and related PFC activation. We found that, only for children with higher levels of irritability, better cognitive flexibility task performance correlated with more PFC activation, consistent with previous research [18]. Furthermore, children with lower levels of irritability exhibited an insignificant association between cognitive flexibility task performance and PFC activation. This finding was consistent with previous research [20,21], which found no association between PFC activation and behavioral task performance. These studies only recruited “typically-developing” subjects, and the insignificant associations might possibly be due to a lack of focus on personality and individual differences. Moreover, highly irritable children have been demonstrated to recruit more PFC activation during the cognitive flexibility task [18]. Thus, the association between PFC activation and the cognitive flexibility task performance might be stronger for children high in irritability than that for children low in irritability. However, we failed to find a significant association between Stroop performance and irritability (*p* > 0.05) in the current sample. This is possibly due to the cognitive flexibility task being easy enough that even highly irritable young children in the current study could engage their PFC recruitment to perform as well as their peers with lower levels of irritability. Thus, we suggest that future research should regard individual differences in personality as an additional variable (i.e., a moderator) in exploring the relationship between behavioral task performance and concurrent brain functioning.

Previous studies have linked irritability in children to cognitive functioning and its neural underpinnings. For example, a previous study found that increased whole brain activations during the inhibitory control task was correlated with relatively higher levels of irritability [65]. Similarly, another study found that more PFC activation during a Go/No-go task was related to increased child irritability [60]. Both studies uncovered a bivariate relationship by simply correlating irritability (i.e., a questionnaire measure) to the brain functioning data during a behavioral task. However, these studies did not construct a complex model from an integrative view by taking behavioral task performance into consideration. Our study, using a three-variables analysis (i.e., a moderation model), investigates the complex affective–cognitive interaction mechanism at the neural level. Moreover, our study serves as evidence of understanding that individual differences in personality have an influence on cognitive performance and are related to the cortical arousal system, which was proposed in Eysenck’s personality model [66,67]. Thus, the current study contributes to our understanding of the interplay between the affective and cognitive processes from a new perspective.

It is important for researchers to consider the relationship between irritability and neurocognitive functioning across a spectrum of irritability severity rather than comparing irritability-related psychiatric disorders with healthy controls. Thus, recent researchers have started to investigate the association between PFC activations and the varying levels of irritability [33,38]. The current study recruited subjects both from the community and clinics to obtain the ratio of full normal to abnormal spectrum of irritability. Our results suggest that highly irritable children might benefit from intervention programs aiming to promote executive function, consistent with previous research [44,45] aiming to ameliorate psychological adjustment (such as emotional competence and externalizing problems) by training executive functions. Thus, clinicians and educators could offer children meaningful opportunities to participate in executive function training activities, which have beneficial effects on alleviating irritability symptoms.

Despite the strengths, some limitations of the current study should be noted. First, since fNIRS is a region-of-interest approach driven by a specific hypothesis and could only detect neural activation of cortical surfaces, the current study identified only the activation of the outer cortex in the PFC. Thus, other neural circuits related to irritability or cognitive flexibility, but distributed in the ventral or medial part of the cortex such as the amygdala or striatum [68], were not able to be monitored in this study. Future studies could use magnetic resonance imaging to investigate the brain activity associated with subcortical areas and functional and structural connectivity to delineate the pattern of brain connectivity associated with irritability. Second, our inference that irritability might play a moderating role in the association between cognitive flexibility task performance and related brain activation could only be limited to the current cross-sectional design. Other moderators, which highlighted different aspects of the relationship between cognitive performance and PFC activations, should be investigated, and a longitudinal design would be preferred in future studies. Third, the measurement of irritability was based on a parent-compiled questionnaire, which is subject to bias. In future studies, it would be necessary to utilize a self-reported or performance-based measure to evaluate child irritability. Fourth, we did not keep a record of the clinical-recruited children’s treatment as their problem may have been caused by undiagnosed medical conditions, which, in turn, may influence irritability and/or cognitive flexibility. Future research should identify the clinical records and some other related information about the child. The present study is, however, a relatively large-sampled neuroimaging study conducted in children at a young age. It is amongst the first to explore the moderating role of irritability in the association between cognitive flexibility task performance and PFC activation from a perspective of variability in affective–cognitive interaction at the neural level.

## 5. Conclusions

In conclusion, we found that young children who responded faster during the cognitive flexibility task recruited more PFC activation. In addition, such an association was stronger for children with higher levels of irritability. Our results proved that irritability and cognitive flexibility might be intertwined at the neural level during early childhood. This study puts forward a novel affective–cognitive interaction perspective to investigate the associations between the cognitive task performance and their neural underpinnings. These results encourage the exploration of EF training effects on children with high irritability.

## Figures and Tables

**Table 1:** The multivariate regression model predicting PFC activation.

Variables	*B*	*SE*	*t*	*p*	95% Confidence Interval for *B*	
					Lower Limit	Upper Limit
Constant	0.04	0.10	0.39	0.690	−0.15	0.23
Stroop RT	−0.35	0.10	−3.65	0.000	−0.54	−0.16
Irritability	0.28	0.10	2.81	0.006	0.08	0.48
Stroop RT × Irritability	−0.22	0.11	−2.11	0.038	−0.43	−0.01
	*R*^2^ = 17.37%, *F* (3,94) = 6.58, *p* < 0.001					

## Data Availability

The data sets used and analyzed in the current study are available from the corresponding author upon reasonable request.
